# Innovative Green Way to Design Biobased Electrospun Fibers from Wheat Gluten and These Fibers’ Potential as Absorbents of Biofluids

**DOI:** 10.1021/acsenvironau.1c00049

**Published:** 2022-01-21

**Authors:** Faraz Muneer, Mikael S. Hedenqvist, Stephen Hall, Ramune Kuktaite

**Affiliations:** † Department of Plant Breeding, Swedish University of Agricultural Sciences, Box 190, SE-23422 Lomma, Sweden; ‡ Fiber and Polymer Technology Department, KTH Royal Institute of Technology, SE-100 44 Stockholm, Sweden; § Solid Mechanics, Lund Institute of Advanced Neutron and X-ray Science (LINXS), Lund University, Box 117, SE-221 00 Lund, Sweden

**Keywords:** gluten proteins, electrospinning, microfibers, protein structure, absorbents, blood absorption, medical textiles

## Abstract

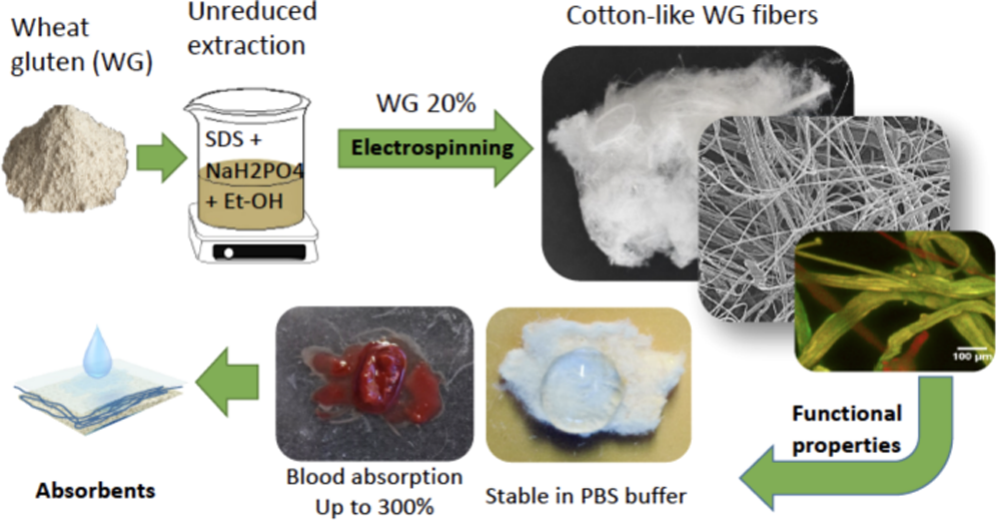

In this study, a new method was developed to successfully design sustainable microfibers from wheat gluten proteins using a nonreducing solvent and electrospinning. We explored the morphology by X-ray tomography, scanning electron microscopy (SEM), and confocal laser scanning microscopy (CLSM), protein chemistry and cross-linking by size exclusion-high-performance liquid chromatography (SE-HPLC), and secondary structure by Fourier transform infrared spectroscopy (FT-IR) of fibers containing 15 and 20% of gluten. The impact of heat (130 °C) post-treatment on the polymerization properties of fibers and their absorption performance in different biofluids were also evaluated. The fibers with 20% gluten showed a uniform architecture supported by a relatively stronger fibrous network as compared to irregular and brittle fibers from 15% gluten. Heat treatment of fibers increased the protein cross-linking in all electrospun fibers as compared to the non-heat-treated fibers, as evidenced by SE-HPLC. An increase in the amount of α-helices and random coils was observed in the proteins of all of the heat-treated fibers compared to the nontreated fibers by FT-IR. This suggested that the heat treatment contributed positively to the gluten protein’s chemical rearrangements, e.g., aggregation, new hydrogen and isopeptide bonding, and conversion of some of the sulfhydryl groups into disulfide cross-links, contributing positively to the functional performance. The heat-treated electrospun fibers with 20% gluten showed a very attractive blood absorption capacity (323%) and reasonable stability in phosphate-buffered saline (PBS) buffer compared to 15% gluten fibers and non-heat-treated fibers. Cotton-like fiber architecture, high blood absorption capacity, and reasonable stability in PBS buffer are properties desired for absorbents of biofluids and should be further explored in healthcare and medical applications.

## 1 Introduction

Micro-/nanofibers produced from a vast majority of synthetic polymers are widely used in medical applications such as tissue engineering and wound dressings, and for drug delivery.^1−4^ These micro-/nanofibrous materials, when serving as absorbents and wound dressings, are required to have specific functional properties, e.g., high surface area, porosity, and suitable mechanical performance.^5,6^ In addition, for absorption of biofluids in various healthcare products, there is a growing demand for biobased, sustainable, safe, and functional materials produced from natural polymers.^7,8^ This is due to the fact that biopolymers offer a good biocompatibility and biodegradability, as well as the advantageous ability to absorb biofluids, which are properties well suited for absorbent applications.^1,5,6,9^

Among natural polymers, wheat gluten (WG) protein is a highly diverse polymer with an inherent complex macromolecular structure and exceptional functional characteristics, e.g., extensibility and strength for various material applications.^10−14^ Wheat gluten protein consists of low-molecular-weight (monomeric) gliadins, and low- and high-molecular-weight (polymeric) glutenins, which provide wheat gluten its unique viscoelastic and strength properties.^15,16^ Few recent studies have shown the successful use of gluten’s viscoelastic properties and molecular structures in producing superabsorbent materials,^7^ porous foams,^8,17^ strong dense composite materials,^14,18^ and electrospun fibers.^19,20^ Electrospun fibers have a high surface area to volume ratio and a large volume of interconnected pores that facilitate good liquid sorption, oxygen transport, and wound healing, and therefore they are preferred for absorbing applications.^21^ However, electrospinning of wheat gluten into micro-/nanofiber materials for absorbent applications has so far been very limited. One of the reasons is the difficulty in dissolving the gluten protein in polar solvents commonly used in electrospinning, which is mainly due to the presence of a high-molecular-weight glutenin fraction with a high degree of cross-linked proteins.^9,22^ In previous studies, reducing conditions (dithiothreitol, DTT, up to 8 M urea solution or 1,1,1,3,3,3-hexafluoro-2-propanol) were used to unfold the gluten proteins and break the existing disulfide linkages to bring the molecular weight and viscosity in the suitable range for electrospinning, also bringing several disadvantages to the protein.^19,20,22,23^ Among these are a strong tendency of contamination (traces of toxic reducing agents) of the fibers, limiting their use as absorbents, wound dressings, and medical textiles. Also, in some cases, when gluten proteins were blended with synthetic polymers for successful electrospinning of nanofibers, it made the fibers nonsustainable.^22,24,25^ Therefore, there is a need to develop suitable methods to produce biobased electrospun fibers as “green” as possible, without chemical contaminants and with suitable functional properties for their potential use as absorbents and medical textiles.

The objective of this study was to produce electrospun fibers from wheat gluten protein using nonreducing solvents and evaluate their gluten fiber potential to be used as absorbents and medical textiles. The structural morphology of the gluten fibers was studied with the aim of increasing the understanding on gluten protein polymerization and molecular structural changes taking place in proteins during electrospinning in relation to absorbing behavior. In addition, the impact of protein concentration on fiber morphology, and that of the post-processing heat treatment on the functional properties of the fibers were also studied.

## 2 Materials and Methods

### 2.1 Materials

The commercial WG powder containing protein 77.7% (with 5.7 conversion factor), starch 5.8%, moisture 6.9%, and fat content 1.2% was purchased from Lantmännen Reppe AB, Lidköping, Sweden. Sodium phosphate monohydrate (NaH_2_PO_4_·H_2_O), sodium dodecyl sulfate (SDS), and ethanol (70 and 96% vol.) were purchased from VWR, Sweden. Defibrinated sheep blood was purchased from Håtunalab AB, Sweden.

### 2.2 Experimental Section

#### 2.2.1 Extraction Solution Preparation and Electrospinning of Fibers

Two WG powders of protein concentrations 15 and 20% (w/w %), respectively, were dissolved in a 50/50 (v/v %) solution of sodium phosphate buffer (2% SDS, 0.5 M NaH_2_PO_4_·H_2_O_,_ pH 6.9) and ethanol (96%). Gluten powder samples were stirred with the extraction solution for at least 2 h, followed by sonication (42 MHz) in a Branson 2510 sonicator for 30 min in a water bath. Thereafter, the samples were centrifuged for 15 min at 10 000 rpm in a Sorvall RC 6+ (Thermo Scientific), and the supernatant was collected and used for electrospinning. The supernatant accounted for ca. 60% of the total WG powder used. To produce electrospun fibers, solutions of WG were pushed through a 10 mL syringe using a syringe pump (Thermo Scientific, SAGE Orion 362). The positive terminal of the high-voltage supply (Spellman SL300) was connected to a metal blunt needle (1 mm gauge opening), and the negative terminal was connected to a collection target (aluminum foil). Thereafter, a voltage of 15–20 kV was used, and the target (collector) was placed at a predetermined distance of 10 cm and at a constant flow rate of 0.5–1 mL/h. The produced WG protein fibers were removed from the collector and stored in an airtight container at room temperature until further analysis. The electrospun fiber samples produced, containing 15 and 20% of WG proteins, were designated as WGF15 and WGF20, respectively.

#### 2.2.2 Scanning Electron Microscopy (SEM)

The electrospun fibers were sputter coated for 40 s with gold particles using a 20 mA voltage in a Cressington 108 auto sputter coater prior to SEM analysis. Thereafter, the coated samples were placed under a scanning electron microscope (Hitachi, SU3500, Japan) and studied at different magnifications using an acceleration voltage of 5 kV.

#### 2.2.3 Confocal Laser Scanning Microscopy (CLSM)

The microfibers’ protein autofluorescence was used to explore the surface and interaction of fibers using a Zeiss LSM880 confocal laser scanning microscope (Carl Zeiss Microscopy) fitted with a 20× LD LCI Plan-Apochromat 1.2 water immersion AutoCorr Dic objective. The imaging was focused on protein autofluorescence excited using 488 and 561 nm lasers, and the emission was recorded from 493–531 to 661–701 nm spectra intervals.

#### 2.2.4 X-Ray Tomography

To determine the structural morphology of the fibers, X-ray tomography was performed using a Zeiss XRadia XRM520 X-ray tomograph at the four-dimensional (4D) Imaging Lab, Lund University, Sweden. The X-ray tomograph was equipped with a polychromatic cone beam that was run at a voltage of 40 kV and a power of 3 W, and the sample exposure time was 4 s. Radiographs were acquired over 360° across the sample. The optical magnification was 4×, resulting in an image width and height of 1014 pixels, in which the pixel size accounted for 1.5014 μm. The inner structure and three-dimensional (3D) structure of the fiber specimen were calculated by defining a region of interest that encompassed the adequate threshold ratio to delineate the internal structure of the gluten fiber cluster, followed by the volume fraction operation of the voxel count plug-in to Image J.

#### 2.2.5 Protein Polymerization Evaluation by Size Exclusion-High-Performance Liquid Chromatography (SE-HPLC)

For evaluation of the protein polymerization and molecular size distribution, SE-HPLC analysis was performed on electrospun protein fibers according to Muneer et al.^14^ with some modifications. This method consisted of a three-step extraction procedure in which fiber samples were dissolved in a buffer during the first extraction, followed by two continuous extractions using the same buffer and additional different sonication intervals. The amount of extracted/solubilized WG protein from the samples was taken as a measure of WG protein polymerization among different samples.

For analysis, 5 (±0.05) mg of each sample was dissolved in 1.4 mL of extraction buffer (0.5% SDS, 0.05M NaH_2_PO_4_·H_2_O, pH 6.9) in a 1.5 mL Eppendorf tube. Tubes with the buffer and sample were shaken for 10 s in a Whirli Vib 2 (Labassco, Sweden) at full speed and thereafter shaken for 5 min in an IKA Vibrax VXR (IKA, Germany) at 2000 rpm. Thereafter, samples were centrifuged at 12 500 rpm for 30 min at room temperature, and the supernatant was collected as first extraction (1Ex). For the second extraction (2Ex), the extraction buffer was added to the pellet from 1Ex and sonicated for 30 s in a Sonyo Soniprep 150 Ultrasonic Disintegrator (Tamro, U.K.), centrifuged, and the supernatant collected was designated as 2Ex. For the third extraction (3Ex), pellet from the 2Ex was filled with the buffer, and this time two sonication intervals (30 + 60 s) were used. During each sonication interval, the samples were left to cool at room temperature to avoid overheating. Thereafter, the samples were centrifuged and the supernatant was collected in HPLC vials.

All three extractions (1Ex, 2Ex, and 3Ex) were analyzed with a Waters 2690 Separation Module, and chromatograms were obtained using a Waters 996 Photodiode Array Detector (Waters) at a wavelength of 210 nm. For analysis, a 20 μL volume was injected into an SE-HPLC column (Bioep-SEC-S 4000, Phenomenex) at an isocratic flow of 0.2 mL/min (50% acetonitrile, 0.1% trifluoroacetic acid, TFA; 50% H_2_O, 0.1% TFA) for a total runtime of 30 min. Chromatograms representing the gluten protein’s molecular profile, amount, and size distribution were integrated and divided into two groups, polymeric proteins (retention 7–14 min) and monomeric proteins (MP) (14–28 min). Triplicates were used for this analysis, and the data was normalized to the WG powder sample.

#### 2.2.6 Fourier Transform Infrared Spectroscopy (FT-IR)

The electrospun WG protein fibers were dried for at least 72 h using silica gel prior to FT-IR spectroscopy analysis. The analysis was carried out using a Spectrum 2000 FT-IR spectrometer (Perkin-Elmer Inc.) equipped with a single-reflection ATR (Golden Gate, Speac Ltd.). A scanning resolution of 4.0 cm^–1^ and data from 32 scans were obtained between 4000 and 600 cm^–1^. Fourier self-de-convoluted curves were obtained with the Spectrum software using an enhancement factor (γ) and smoothing factor of 2 and 70%, respectively. A Savitzky–Golay 5-point second-order derivative analysis was done to find the underlying corresponding peaks in order to determine the specific secondary structure.^13,26^ Amide I band was baseline corrected and peak fitting was performed for 8–9 Gaussian peaks using the Fityk software.^27^

#### 2.2.7 Heat Treatment of Wheat Gluten Fibers

For studying the water affinity and improving the stability of the electrospun gluten fibers, heat treatment was applied on the fiber samples. The annealing procedure was performed at 130 °C for 2 h in a laboratory oven (UF110, Memmert, Germany). Thereafter, the samples were removed from the oven and stored in an airtight container at room temperature until further analysis. The heat-treated electrospun fibers produced with the 15 and 20% gluten proteins were designated as T-WGF15 and T-WGF20, respectively.

#### 2.2.8 Absorption and Stability of the Fibers in Liquids

For analysis of the absorption of defibrinated sheep blood, the WG fibers were weighed in duplicates prior to the test. Defibrinated sheep blood was used, and the drops of blood were applied on the surface of the gluten fibers with a pipette up to three times the weight of the fiber sample. Thereafter, the gluten fiber samples with blood droplets were left for at least 1 h at room temperature, and afterwards, the fiber specimens were removed from the Petri dish and placed on a paper towel to remove the excessive non-absorbed blood prior to weighing. Two replicates were used for each sample.

For analysis of the stability and absorption of gluten fibers in phosphate-buffered saline (PBS) solution, in the first step, a drop of PBS buffer (pH 7.4) was poured on the surface of both heat-treated and nontreated gluten fibers, and the absorption of the droplet was visually evaluated, e.g., whether the droplet was absorbed within the given time or showed a lotus effect. In the second step, the gluten fiber’s stability in PBS aqueous environment was evaluated, and the whole sample was immersed in a PBS buffer and kept overnight. The overall visual morphology of the samples was evaluated, and pictures taken after 1 h after immersion were included in this study.

## 3 Results and Discussion

### 3.1 Production and Characterization of Gluten Fibers from Nonreducing Solutions

#### 3.1.1 Production

**Figure 1:**
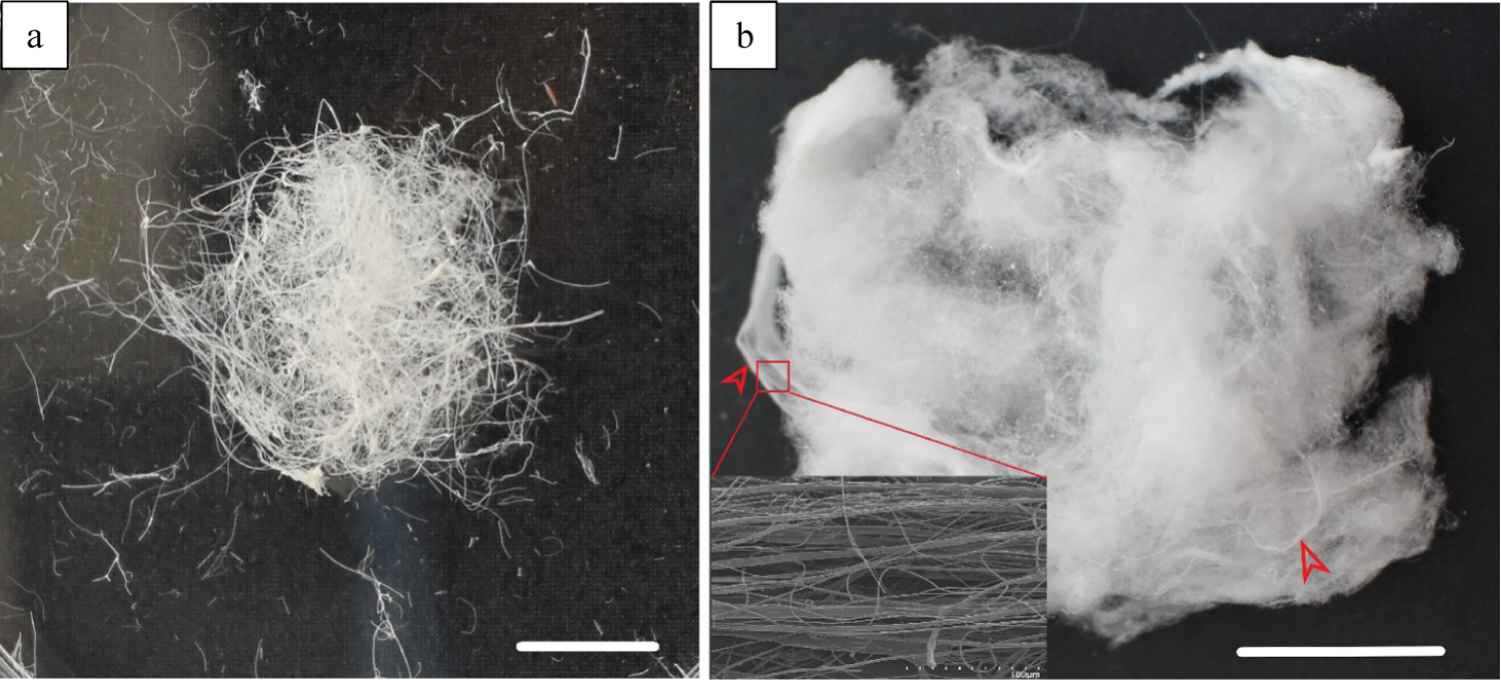
Representative images of electrospun fibers from diverse gluten protein concentrations: (a) WGF15 and (b) WGF20 (scale bar is 1 cm); red arrows indicate fiber bundles; the inset SEM figure shows unidirectionally aligned fibers.

Wheat gluten proteins are complex large molecules that have been electrospun into fibers in reducing solutions,^22,28^ through selectively solubilizing the monomeric protein fractions and excluding the disulfide-bound part of the polymer. Similarly, in another study, 1,1,1,3,3,3-hexafluoro-2-propanol (HFIP) was used to solubilize and electrospin the gluten protein.^20^ In this study, we used a 50/50 (v/v %) solution of sodium phosphate buffer (2% SDS, 0.5 M NaH_2_PO_4_·H_2_O, pH 6.9) and ethanol (96%) combined with sonication (42 MHz) treatment to dissolve wheat gluten proteins and electrospin into cotton-like fibers, as shown in Figure 1. Two different types of fibers were electrospun from 15 and 20% gluten protein concentrations in the solutions (WGF15 and WGF20, respectively). The WGF15 electrospun fibers were rather disorganized, fragile, and brittle, while the WGF20 fibers were flexible, rather continuous, and relatively resistant for manual handling, e.g., more desirable in terms of their appearance and resemblance to cotton, with some fibers unidirectionally aligned in fibrous bundles (Figure 1b, highlighted by red arrows; inset, SEM micrograph).

#### 3.1.2 Morphology

**Figure 2:**
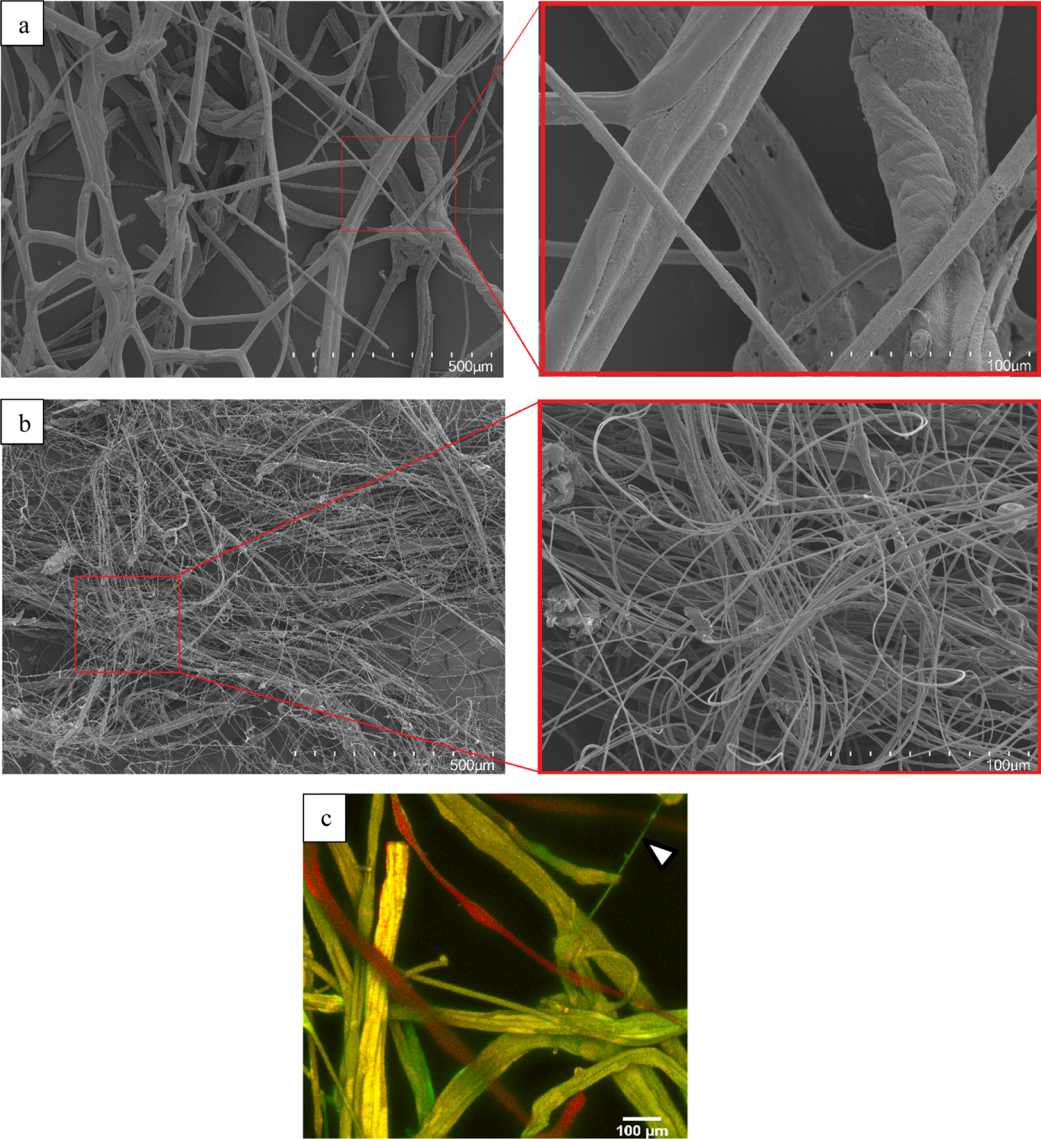
Scanning electron micrographs of electrospun fibers from diverse gluten protein concentrations, WGF15 (a) and WGF20 (b), and CLSM autofluorescence image of WGF15 fibers (c) indicating nanosized fibers (white pointer).

The SEM micrographs of WGF15 and WGF20 fibers showed two fibrous networks with greatly varying fiber morphologies (Figure 2). The lower gluten concentration (15%) yielded mainly a larger diameter and more agglomerated fibers (fibers fused in groups in WGF15; Figure 2a, enlarged picture) compared to WGF20, suggesting that fiber size (thickness) distribution was largely dependent on the gluten protein concentration of a spinning dope. The WGF15 fiber’s diameter ranged ca. 70–100 μm and showed an uneven surface morphology as well as fragmentation as compared to WGF20 fibers, being rather continuous with very few beads as observed by SEM (compare Figure 2a with b). Besides, the formation of nanofibers in WGF15 was also evidenced by CLSM (Figure 2c, white pointer). One of the reasons for the major larger diameter of WGF15 samples is the adhesion of multiple fibers into bundles on different length scales, as shown in Figure 2a (inset). A phenomenon of fusion of fibers was observed during the spinning process, which resulted in the majority of fibers forming bundles at the needle exit before finally reaching the target.

The WGF20 samples showed a randomly arranged uniform-sized (ca. 5–10 μm) electrospun fibrous network (Figure 2b). In general, a higher amount of fibers were obtained for WGF20 than WGF15 in similar spinning times. The higher concentration of gluten proteins in the solution (i.e., 20%) suggested the availability of a higher amount of unfolded spinnable proteins. This offered a higher probability for molecular-chain entanglements between the gluten proteins, which potentially led to a smooth spinning operation, resulting in a higher amount of fibers, as observed previously in aqueous electrospinning of wheat gluten proteins.^22^ A similar cotton-like electrospun fibrous material was obtained in a previous study where glutenins were used to produce electrospun fibers, although reducing agents were used to break the disulfide cross-links to obtain a spinnable gluten protein solution.^9^ The WG fibers produced in this study showed a distinctive round fiber morphology as compared to the ribbon-like flat fiber morphology in wheat gluten electrospun fibers shown in some of the previous studies.^20,24,25^ A blend of gluten and soy proteins with addition of poly(vinyl alcohol) (PVA) also showed a round fiber morphology similar to the wheat gluten fibers produced in this study.^29^ Similarly, soy protein and polyethylene oxide blends produced fibers with round fiber morphology, improved spinnability, and air filtration properties.^30^

**Figure 3:**
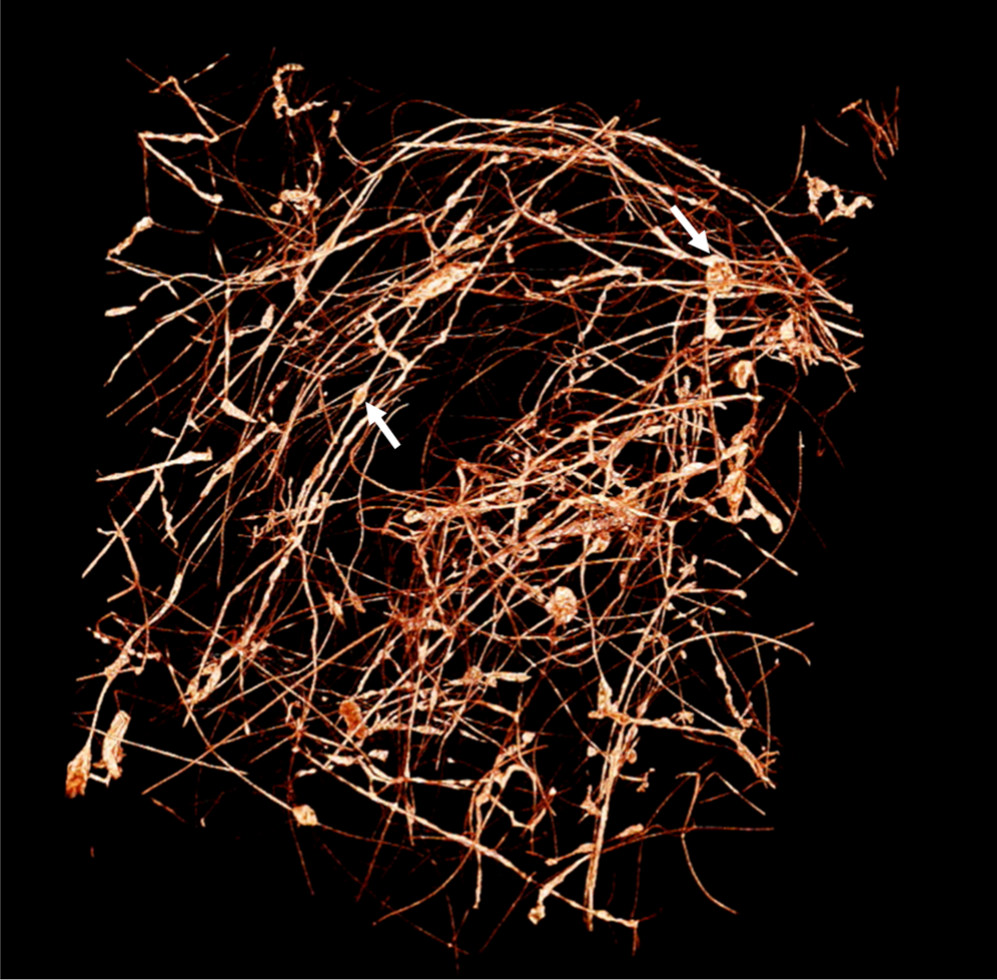
X-ray tomography of the electrospun fibers of WGF20; arrows indicate few irregularities of fiber thickness.

X-ray tomography was used to study the internal 3D structure, as it has been used previously to study the internal structure of cereal seeds and biobased foams.^31−33^ In the WGF20 fibrous 3D structure, some beads or knot-like structures were also observed, which were intermittently spread among the fibrous 3D network, as was shown by X-ray tomography (Figure 3). However, these beads were different from the typical “bead-on-string” structure, where beads were a part of the fiber morphology as shown in a WG/PVA composite fiber.^22^ The presence of these bead structures in this study could be explained by the fact that during electrospinning, the volatile solvent (as EtOH) evaporates and proteins form a film clogging the opening of the needle. When the high voltage and pressure from the pump overcome the resistance created by the film, the protein solution flies to the target in the form of a misty spray and dries as small particles. Such a phenomenon can be avoided with a continuous thorough cleaning of the surface of the needle every few minutes to stop film formation around the syringe opening (which in our case disturbed the electrospinning).

We conclude that at 20% gluten concentration of the spinning solution, the fibers were uniform and very much alike cotton fibers with few beads and rather continuous fibers, which were randomly arranged and in some cases well aligned into fibrous bundles.

### 3.2 Protein Polymerization Behavior in Protein Spinning Solutions and Fibers

**Figure 4:**
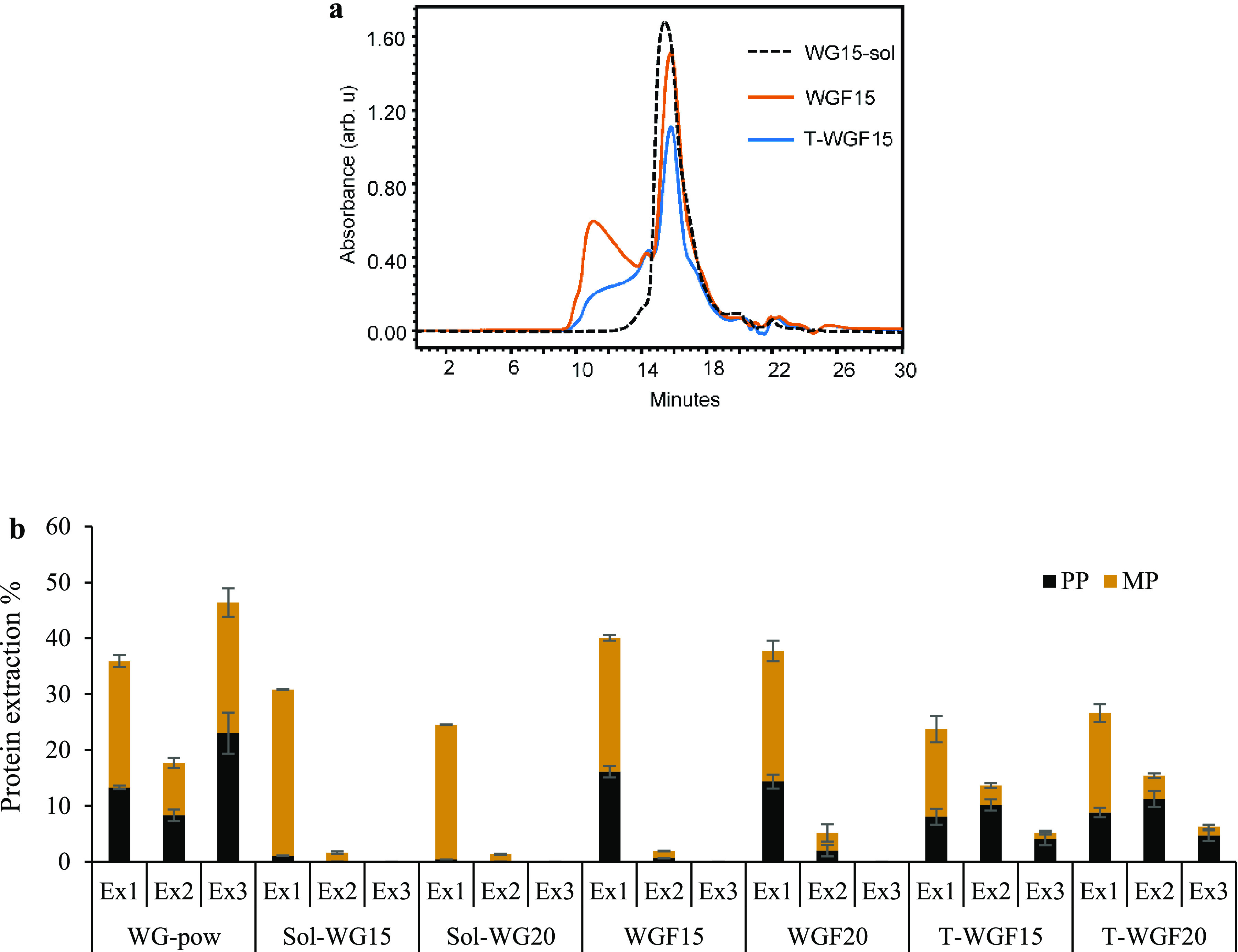
Representative SE-HPLC chromatograms of first extraction (Ex1) of gluten protein spinning solutions, electrospun fibers (WGF15), and heat-treated fibers (T-WGF15) showing the protein’s solubility profile (a), and polymeric and monomeric proteins’ solubility of WG protein powders, electrospinning solutions, and electrospun fibers (b).

SE-HPLC analysis was performed to analyze the protein solubility in both heat-treated and nontreated gluten electrospun fibers, which was taken as a measure of the protein polymerization behavior among the studied samples (Figure 4). A representative SE-HPLC chromatogram showed a decreased protein solubility of both polymeric (PP) and monomeric (MP) proteins in heat-treated fiber samples compared to nontreated fibers (Figure 4a; compare WGF15 with T-WGF15). For WG protein solutions (used for electrospinning), only a large amount of MP fraction was extracted for both WG-sol 15 and WG-sol 20 (Figure 4b). Extraction of only the MP fraction can be explained by the fact that during the preparation of the spinning solution, the extraction buffer (2% SDS, 0.5 M NaH_2_PO_4_, ethanol 96%) dissolved most of the gliadins (due to the presence of 96% ethanol (50% v/v)) and only some of the glutenins, due to the presence of SDS and sonication treatment (larger polymeric proteins broke into smaller polymeric proteins), respectively (the supernatant was used for electrospinning the fibers and for SE-HPLC analyses).

In both WGF15 and WGF20 fiber samples, heat-treated fibers showed a higher degree of protein cross-linking as compared to nontreated fibers, as was shown by the lower protein solubility of both PP and MP in the 1Ex followed by extraction of proteins upon sonication in 2Ex and 3Ex steps (Figure 4b). A high degree of protein cross-linking has been previously reported at temperatures of 110 and 130 °C in compression molded and extruded wheat gluten protein films and composite materials.^14,34−37^ In both types of fibers, i.e., WGF15 and WGF20, almost all PP and MP were extracted during the 1Ex, indicating that the fiber formation was a result of protein cross-linking, although increased protein chain entanglements were possibly maintained by hydrogen bonding and other weak interactions (almost no pellet was left after the 1Ex). However, in the heat-treated fibers (T-WGF15 and T-WGF20), the lower amount of PP and MP extracted during the 1Ex explains that heat treatment of fibers at 130 °C induced a certain degree of protein cross-linking and promoted the formation of protein aggregates maintained by intermolecular disulfide cross-links and isopeptide bonds (Figure 4b). Extraction of SDS-insoluble proteins upon sonication results in breaking of additional disulfide cross-links between the gluten proteins,^14,38^ whereas weaker protein interactions are already broken by SDS treatment during the 1Ex.^39^

The wheat gluten proteins exist as coiled or folded chains that are stabilized via disulfide bonds between the cysteine residues and other weaker protein interactions, e.g., ionic interactions.^40^ However, when proteins are in solution, stretching and disruption of the weaker bonds takes place.^20,25^ When immediate removal of the solvent during electrospinning occurs, the proteins potentially re-form intramolecular and intermolecular cross-links between the peptide chains, resulting in the formation of fibers.^22^ The heat treatment of fibers, as was done in this study, is known to promote the formation of disulfide cross-links between polymer peptide chains and stabilize the fibers, as reported previously for reduced wheat gluten protein electrospun fibers.^20^ The formation of intermolecular disulfide cross-links and isopeptide bonding in thermally processed proteins and their positive impact on the functional properties of materials have been reported previously in whey proteins nanofibrils,^41^ gluten-based foams,^42^ and films.^43^

### 3.3 Gluten Protein Secondary Structure of Electrospun Fibers

**Figure 5:**
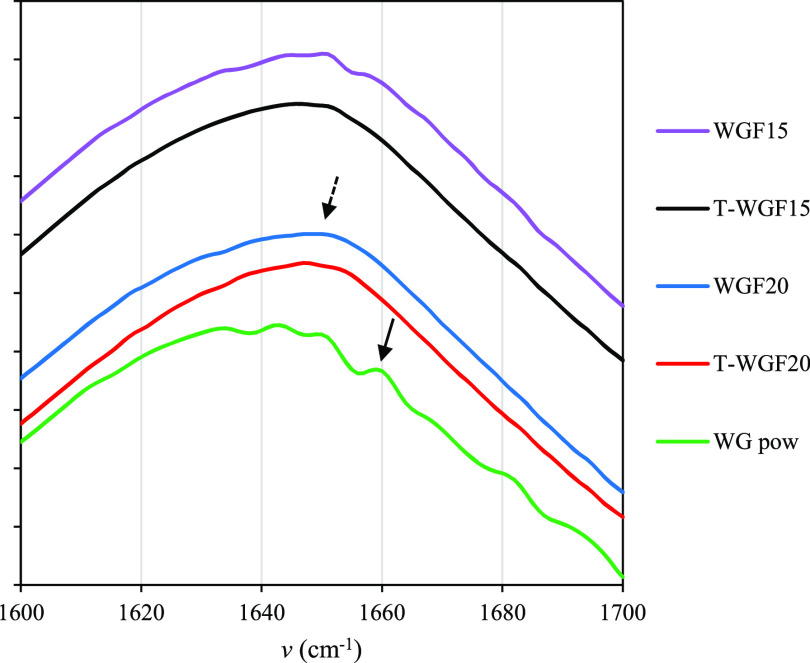
Protein secondary structure profile of the WG powder and electrospun fibers and heat-treated fibers.

FT-IR spectroscopy was performed to analyze the protein secondary structure. The results of the amide I region (1600–1700 cm^–1^) of WG powder, and heat-treated and nontreated electrospun gluten fibers are presented in Figure 5. From the IR spectrum, the relative amounts of α-helices/random coils and β-sheet structures were determined by comparing the relative size/intensity of peak shoulders in the regions 1645–1660 and 1620–1635 cm^–1^, respectively.^39^ For all of the fiber samples, the high-intensity peak in the region 1645–1660 cm^–1^ showed the presence of α-helices/random coiled structures (Figure 5, dotted arrow), as compared to the pristine WG powder, where only two low-intensity peaks related to the α-helices/random coiled structures in the 1650–1660 cm^–1^ region were observed (Figure 5, solid black arrow). This indicated that electrospinning of gluten protein into fibers and post-heat treatment of fibers contributed to the conversion of some of the unordered structure of the protein to α-helices/random coils; hence, an overall more organized structure was maintained by the hydrogen bonding and disulfide cross-links between the protein polypeptide chains, as has been observed from the SE-HPLC results (Figure 4). The peak shoulder/intensity between 1620 and 1635 cm^–1^ representing the β-sheets was very similar for all of the WG fibers and pristine WG powder samples (Figure 5). This suggests that during the preparation of protein solutions, electrospinning of solutions into fibers, and their post-heat treatment, the proteins could preserve the β-sheet conformation, as was previously reported for WG electrospun fibers.^19^

Due to very small differences in the FT-IR spectrum of all the WG fibers and heat-treated fiber samples, peak fitting was performed for each spectrum to study the underlying structural peaks (Supporting Information; Table S1 and Figure S1). In any of the WG fiber samples, the β-sheet structural peaks did not largely shift for both normal and heat-treated samples (Figure S1). However, heat treatment of WG fibers at 130 °C increased the α-helices and random coil structures, as can be seen by the shifting of the peak at 1644 cm^–1^ (Figure S1a) to 1650–1660 cm^–1^ (Figure S1b) for WGF15 vs T-WGF15 samples. A similar phenomenon was observed for the WGF20 sample, where the intensity of the peak at around 1640 cm^–1^ decreased upon heat treatment and formation of a new high-intensity peak was observed at 1660 cm^–1^ (Figure S1d, formation of α-helices/random coils). The transformation of unordered structural conformation upon annealing has previously been reported in reduced wheat gluten electrospun fibers.^23^ This suggests that annealing of fibers at high temperature (130 °C) contributed to protein aggregation, new hydrogen bonding, and conversion of some of the free sulfhydryl groups into disulfide cross-links.^44^ The FT-IR results of heat-treated fibers complemented the SE-HPLC results in this study (Figure 4) regarding the relative increase in protein aggregation maintained by disulfide cross-links and other noncovalent interactions versus nontreated fiber samples.

### 3.4 Blood Absorption and Stability of Fibers in PBS Buffer

**Figure 6:**
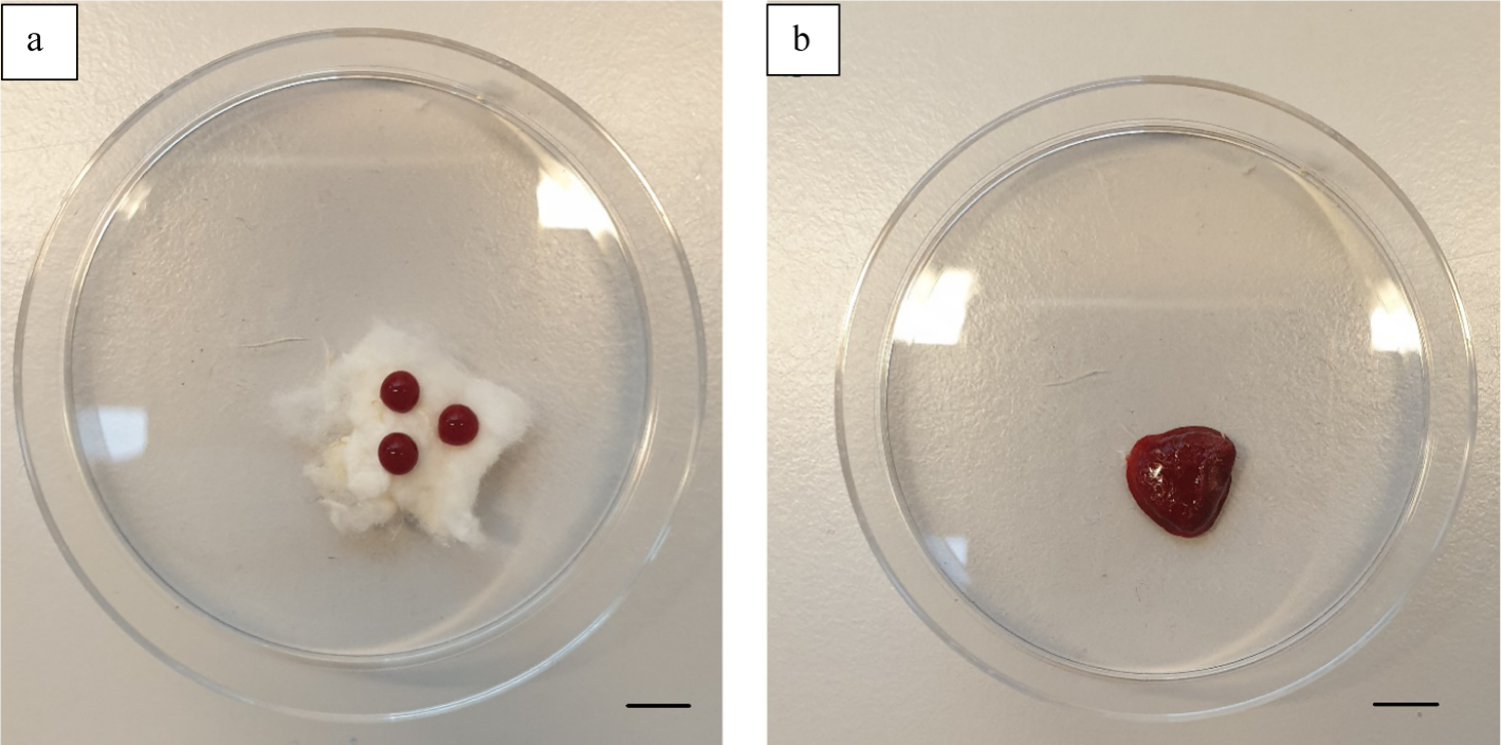
Defibrinated sheep blood absorption of T-WGF20 fiber after 1 min (a) and after 20 min (b).

All of the electrospun fiber samples showed variation in the absorption capacity of defibrinated sheep blood (Table 1). Heat treatment of gluten fibers at 130 °C for 2 h contributed to an increased absorption capacity of the fibers as presented by the T-WGF20 sample, which showed the highest absorption capacity of 323% (±38) after 1 h of immersion in defibrinated sheep blood (Table 1). The blood absorption for the nontreated WGF20 sample was 228% (±9). The heat-treated T-WGF15 sample showed an absorption capacity of 216% (±10), whereas the nontreated WGF15 samples collapsed and were not retrievable after immersion in blood for 1 h. Figure 6a demonstrates that when three drops of blood were dropped on the surface of a representative T-WGF20 sample using a pipette, they stayed on the surface of the fibrous network in the form of droplets for the first 2–3 min. Thereafter, the surface area of the fibrous film started to decrease, and after 20 min, the whole film was soaked in blood (Figure 6b). This blood absorption phenomenon of WG fiber samples can be further studied for absorbant applications, i.e., bandages and medical textiles. However, for successful applications of biopolymer-based medical textiles as wound dressings, functional properties such as strength, flexibility, moisture absorption capacity, and permeability should be achieved.^45^ Heat-induced cross-linking and functionalization of wheat gluten proteins has been done in previous studies to improve the superabsorbent capacity of protein-based materials for applications in personal hygiene products.^42,46−48^ Functionalization (with EDTAD and genipin) of wheat gluten proteins has shown a blood absorption capacity of up to 250%, which is slightly lower than the values observed in this study.^47^ However, the functionalized materials produced in previous studies have shown improved liquid retention and stability properties compared to the wheat gluten fibers produced in this study. This is due to the fact that in all of the functionalized materials, the proteins showed increased cross-linking, and in addition, these proteins contained additional carboxyl groups that helped the proteins to absorb and retain a higher quantity of liquids.^7^ Therefore, to further improve the liquid absorption and retention capacity of electrospun wheat gluten fibers, a similar functionalization and cross-linking of proteins with chemical cross-linkers such as EDTAD and genipin needs to be studied.

**Table 1:** Defibrinated Sheep Blood Absorption Values of the Gluten Fiber Samples^a^

WG fiber samples	average blood absorption %
WGF15	n/a
WGF20	228 (9)
T-WGF15	216 (10)
T-WGF20	323 (38)

a Numbers in brackets represent standard deviation.

**Figure 7:**
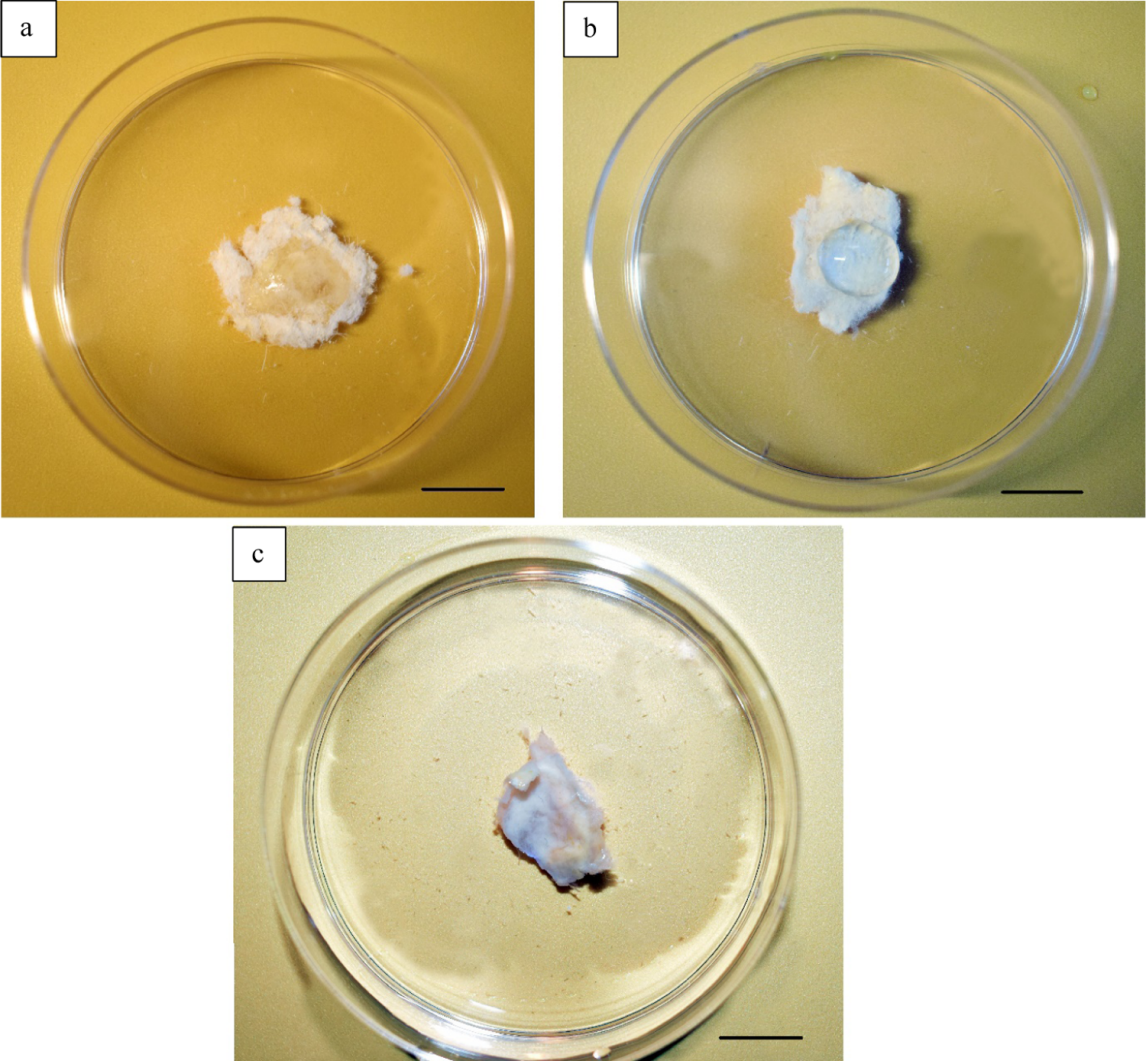
PBS buffer absorption of T-WGF15 (a) and T-WGF20 (b) electrospun fibers where the pictures were taken immediately, and (c) T-WGF20 fibers immersed in PBS buffer solution after 1 h.

Heat-treated fiber films showed variations in liquid absorption behavior and stability in PBS buffer (Figure 7). When a drop of PBS buffer was placed on the surface of the T-WGF15 fiber film, it quickly absorbed the liquid (Figure 7a). However, when a drop of PBS buffer was added to the T-WGF20 fiber film, it showed hydrophobic properties, e.g., lotus effect, with the droplet staying on the surface of film for several minutes before getting absorbed by the film (Figure 7b). When the fibrous networks were completely immersed in the PBS buffer, the T-WGF20 fiber films were stable (Figure 7c), whereas the T-WGF15 fiber films disintegrated immediately after full immersion in the PBS buffer solution (Supporting Information, Figure S2).

An increase in the water stability properties of WG electrospun fibers due to post-heat and microwave treatment was also reported in previous studies.^9,23^ Heat treatment promotes the conversion of the sulfhydryl group (−SH) to disulfide cross-links (−S–S−) to fix the fibers and improve their water stability.^19^ The hydrophobic behavior of T-WGF20 electrospun fibers can be explained by the fact that protein cross-linking during heat treatment unfold the protein chains and expose the hydrophobic residues, which can lead to increased surface hydrophobicity.^23^ The liquid absorption properties and stability of electrospun fibers in liquid growth systems are of critical importance for their use in biomaterials and potential medical applications, e.g., absorbents and wound dressings.

## 4 Conclusions

In this study, a new green and sustainable method combining SDS-phosphate buffer (2% SDS, 0.5 M NaH_2_PO_4_·H_2_O)/ethanol (96%) solution (50/50 v/v) and sonication was developed to successfully design wheat gluten protein fibers using electrospinning. Our study for the first time showed this method as a good alternative to replace environmentally toxic reducing agents (e.g., 1,1,1,3,3,3-hexafluoro-2-propanol, DTT, and higher concentrations of urea (up to 8 M solutions)) to dissolve gluten proteins for electrospinning of fibers. The wheat gluten protein concentration (20%) played a significant role in the electrospun fiber architecture, micro-/nano-morphology, and functional performance. The gluten concentration (20%) contributed to a smooth electrospinning operation and delivered diverse-diameter (from nano to ca. 10 μm) cotton-like fibers. The 20% gluten fibers showed more unfolded proteins in spinning solution and contributed to the formation of new chain entanglements via hydrogen bonding, and inter- and intramolecular protein interactions.

Post-heat treatment (130 °C for 2 h) of electrospun protein fibers played a significant role in inducing a higher degree of protein cross-linking (decreased protein solubility as shown by SE-HPLC) among heat-treated fibers compared to non-heat-treated ones. This was also evident in the secondary structure of the proteins with a relatively higher size/intensity of α-helices/random coils’ peaks in the heat-treated samples compared to the nontreated ones. Heat treatment also contributed to improving the blood absorption capacity and stability in PBS buffer solution of the produced fiber samples. The T-WGF20 samples were able to absorb ca. 100% more sheep blood, and they were more stable in PBS buffer as compared to the non-heat-treated samples.

In conclusion, a gluten protein concentration of 20% in the spinning dope was most favorable for successful production of potentially desirable electrospun fibers. Heat treatment of fibers improved fiber the liquid absorption capacity by increasing the protein aggregation in fibers and increased the stability in the liquid environment by unfolding protein chains and exposing the hydrophobic residues to the surface of the fibers. The blood absorption capacity and water stability properties of gluten protein fibers show potential for medical applications, although their functional properties, e.g., strength, flexibility, and liquid absorption, need to be further explored and improved.

## Supporting Information Available

Details on relative amounts of gluten protein secondary structure of fibers from the de-convoluted absorbance spectra studied by FT-IR (Table S1 and Figure S1) and liquid stability behavior in PBS buffer solution of electrospun fibers are included (Figure S2) (PDF)

## References

[ref1] Abrigo M.; McArthur S. L.; Kingshott P. Electrospun nanofibers as dressings for chronic wound care: advances, challenges, and future prospects. Macromol. Biosci. 2014, 14, 772–792. 10.1002/mabi.201300561. 24678050

[ref2] Maheshwari S. U.; Kumar S. V.; Nagiah N.; Uma T. S. Electrospinning of polyvinylalcohol–polycaprolactone composite scaffolds for tissue engineering applications. Polym. Bull. 2013, 70, 2995–3010. 10.1007/s00289-013-1002-4.

[ref3] Sill T. J.; von Recum H. A. Electrospinning: applications in drug delivery and tissue engineering. Biomaterials 2008, 29, 1989–2006. 10.1016/j.biomaterials.2008.01.011. 18281090

[ref4] Li L.; Li H.; Qian Y.; Li X.; Singh G. K.; Zhong L.; Liu W.; Lv Y.; Cai K.; Yang L. Electrospun poly (ε-caprolactone)/silk fibroin core-sheath nanofibers and their potential applications in tissue engineering and drug release. Int. J. Biol. Macromol. 2011, 49, 223–232. 10.1016/j.ijbiomac.2011.04.018. 21565216

[ref5] Liu G.; Gu Z.; Hong Y.; Cheng L.; Li C. Electrospun starch nanofibers: Recent advances, challenges, and strategies for potential pharmaceutical applications. J. Controlled Release 2017, 252, 95–107. 10.1016/j.jconrel.2017.03.016. 28284833

[ref6] Zhao Y.; Qiu Y.; Wang H.; Chen Y.; Jin S.; Chen S. Preparation of nanofibers with renewable polymers and their application in wound dressing. Int. J. Polym. Sci. 2016, 2016, 4672839 10.1155/2016/4672839.

[ref7] Capezza A. J.; Lundman M.; Olsson R. T.; Newson W. R.; Hedenqvist M. S.; Johansson E. Carboxylated Wheat Gluten Proteins: A Green Solution for Production of Sustainable Superabsorbent Materials. Biomacromolecules 2020, 21, 1709–1719. 10.1021/acs.biomac.9b01646. 31899621

[ref8] Capezza A. J.; Wu Q.; Newson W. R.; Olsson R. T.; Espuche E.; Johansson E.; Hedenqvist M. S. Superabsorbent and Fully Biobased Protein Foams with a Natural Cross-Linker and Cellulose Nanofibers. ACS Omega 2019, 4, 18257–18267. 10.1021/acsomega.9b02271. 31720526 PMC6844118

[ref9] Xu H.; Cai S.; Sellers A.; Yang Y. Intrinsically water-stable electrospun three-dimensional ultrafine fibrous soy protein scaffolds for soft tissue engineering using adipose derived mesenchymal stem cells. RSC Adv. 2014, 4, 15451–15457. 10.1039/c3ra47547f.

[ref10] Kuktaite R.; Ravel C. Wheat Gluten Protein Structure and Function: Is There Anything New under the Sun?. In Wheat Quality For Improving Processing And Human Health; Igrejas G.; Ikeda T. M.; Guzmán C., Eds.; Springer International Publishing: Cham, 2020; pp 9–19.

[ref11] Diuk Andrade F.; Newson W. R.; Bernardinelli O. D.; Rasheed F.; Cobo M. F.; Plivelic T. S.; Ribeiro deAzevedo E.; Kuktaite R. An insight into molecular motions and phase composition of gliadin/glutenin glycerol blends studied by 13C solid-state and 1H time-domain NMR. J. Polym. Sci., Part B: Polym. Phys. 2018, 56, 739–750. 10.1002/polb.24586.

[ref12] Rasheed F.; Plivelic T. S.; Kuktaite R.; Hedenqvist M. S.; Johansson E. Unraveling the Structural Puzzle of the Giant Glutenin Polymer—An Interplay between Protein Polymerization, Nanomorphology, and Functional Properties in Bioplastic Films. ACS Omega 2018, 3, 5584–5592. 10.1021/acsomega.7b02081. 30023922 PMC6045469

[ref13] Muneer F.; Andersson M.; Koch K.; Hedenqvist M. S.; Gällstedt M.; Plivelic T. S.; Menzel C.; Rhazi L.; Kuktaite R. Innovative Gliadin/Glutenin and Modified Potato Starch Green Composites: Chemistry, Structure, and Functionality Induced by Processing. ACS Sustainable Chem. Eng. 2016, 4, 6332–6343. 10.1021/acssuschemeng.6b00892.

[ref14] Muneer F.; Andersson M.; Koch K.; Menzel C.; Hedenqvist M. S.; Gällstedt M.; Plivelic T. S.; Kuktaite R. Nanostructural Morphology of Plasticized Wheat Gluten and Modified Potato Starch Composites: Relationship to Mechanical and Barrier Properties. Biomacromolecules 2015, 16, 695–705. 10.1021/bm5017496. 25629918

[ref15] Wieser H. Chemistry of gluten proteins. Food Microbiol. 2007, 24, 115–119. 10.1016/j.fm.2006.07.004. 17008153

[ref16] Johansson E.; Malik A. H.; Hussain A.; Rasheed F.; Newson W. R.; Plivelic T.; Hedenqvist M. S.; Gällstedt M.; Kuktaite R. Wheat Gluten Polymer Structures: The Impact of Genotype, Environment, and Processing on Their Functionality in Various Applications. Cereal Chem. J. 2013, 90, 367–376. 10.1094/CCHEM-08-12-0105-FI.

[ref17] Wu Q.; Andersson R. L.; Holgate T.; Johansson E.; Gedde U. W.; Olsson R. T.; Hedenqvist M. S. Highly porous flame-retardant and sustainable biofoams based on wheat gluten and in situ polymerized silica. J. Mater. Chem. A 2014, 2, 20996–21009. 10.1039/C4TA04787G.

[ref18] Kuktaite R.; Türe H.; Hedenqvist M. S.; Gällstedt M.; Plivelic T. S. Gluten Biopolymer and Nanoclay-Derived Structures in Wheat Gluten–Urea–Clay Composites: Relation to Barrier and Mechanical Properties. ACS Sustainable Chem. Eng. 2014, 2, 1439–1445. 10.1021/sc500017y.

[ref19] Xu H.; Cai S.; Sellers A.; Yang Y. Electrospun ultrafine fibrous wheat glutenin scaffolds with three-dimensionally random organization and water stability for soft tissue engineering. J. Biotechnol. 2014, 184, 179–186. 10.1016/j.jbiotec.2014.05.011. 24862198

[ref20] Woerdeman D. L.; Ye P.; Shenoy S.; Parnas R. S.; Wnek G. E.; Trofimova O. Electrospun Fibers from Wheat Protein: Investigation of the Interplay between Molecular Structure and the Fluid Dynamics of the Electrospinning Process. Biomacromolecules 2005, 6, 707–712. 10.1021/bm0494545. 15762633

[ref21] Reddy M. M.; Vivekanandhan S.; Misra M.; Bhatia S. K.; Mohanty A. K. Biobased plastics and bionanocomposites: Current status and future opportunities. Prog. Polym. Sci. 2013, 38, 1653–1689. 10.1016/j.progpolymsci.2013.05.006.

[ref22] Dong J.; Asandei A. D.; Parnas R. S. Aqueous electrospinning of wheat gluten fibers with thiolated additives. Polymer 2010, 51, 3164–3172. 10.1016/j.polymer.2010.04.058.

[ref23] Liu C.; Ma X. Study on the mechanism of microwave modified wheat protein fiber to improve its mechanical properties. J. Cereal Sci. 2016, 70, 99–107. 10.1016/j.jcs.2016.05.018.

[ref24] Wang H.; She Y.; Chu C.; Liu H.; Jiang S.; Sun M.; Jiang S. Preparation, antimicrobial and release behaviors of nisin-poly (vinyl alcohol)/wheat gluten/ZrO2 nanofibrous membranes. J. Mater. Sci. 2015, 50, 5068–5078. 10.1007/s10853-015-9059-0.

[ref25] Woerdeman D. L.; Shenoy S.; Breger D. Role of chain entanglements in the electrospinning of wheat protein-poly (vinyl alcohol) blends. J. Adhes. 2007, 83, 785–798. 10.1080/00218460701588398.

[ref26] Muneer F.; Johansson E.; Hedenqvist M. S.; Plivelic T. S.; Markedal K. E.; Petersen I. L.; Sørensen J. C.; Kuktaite R. The impact of newly produced protein and dietary fiber rich fractions of yellow pea (*Pisum sativum* L.) on the structure and mechanical properties of pasta-like sheets. Food Res. Int. 2018, 106, 607–618. 10.1016/j.foodres.2018.01.020. 29579966

[ref27] Wojdyr M. Fityk: a general-purpose peak fitting program. J. Appl. Crystallogr. 2010, 43, 1126–1128. 10.1107/S0021889810030499.

[ref28] Reddy N.; Yang Y. Self-crosslinked gliadin fibers with high strength and water stability for potential medical applications. J. Mater. Sci.: Mater. Med. 2008, 19, 2055–2061. 10.1007/s10856-007-3294-0. 17968503

[ref29] Lubasova D.; Mullerova J.; Netravali A. N. Water-resistant plant protein-based nanofiber membranes. J. Appl. Polym. Sci. 2015, 132, 41852 10.1002/app.41852.

[ref30] Lubasova D.; Netravali A.; Parker J.; Ingel B. Bacterial Filtration Efficiency of Green Soy Protein Based Nanofiber Air Filter. J. Nanosci. Nanotechnol. 2014, 14, 4891–4898. 10.1166/jnn.2014.8729. 24757959

[ref31] Kuktaite R.; Repo-Carrasco-Valencia R.; Cornejo Hurtado de Mendoza C.; Plivelic T. S.; Hall S.; Johansson E. Innovatively processed quinoa (Chenopodium quinoa Willd) food: Chemistry, structure and end-use characteristics. J. Sci. Food Agric. 2021, 11214 10.1002/jsfa.11214. 33709442

[ref32] Ispiryan L.; Kuktaite R.; Zannini E.; Arendt E. K. Fundamental study on changes in the FODMAP profile of cereals, pseudo-cereals, and pulses during the malting process. Food Chemistry 2021, 343, 128549 10.1016/j.foodchem.2020.128549. 33189480

[ref33] Ceresino E. B.; Johansson E.; Sato H. H.; Plivelic T. S.; Hall S. A.; Kuktaite R. Morphological and structural heterogeneity of solid gliadin food foams modified with transglutaminase and food grade dispersants. Food Hydrocolloids 2020, 108, 105995 10.1016/j.foodhyd.2020.105995.

[ref34] Gällstedt M.; Mattozzi A.; Johansson E.; Hedenqvist M. S. Transport and Tensile Properties of Compression-Molded Wheat Gluten Films. Biomacromolecules 2004, 5, 2020–2028. 10.1021/bm040044q. 15360319

[ref35] Sun S.; Song Y.; Zheng Q. Thermo-molded wheat gluten plastics plasticized with glycerol: effect of molding temperature. Food Hydrocolloids 2008, 22, 1006–1013. 10.1016/j.foodhyd.2007.05.012.

[ref36] Kuktaite R.; Plivelic T. S.; Türe H.; Hedenqvist M. S.; Gällstedt M.; Marttila S.; Johansson E. Changes in the hierarchical protein polymer structure: urea and temperature effects on wheat gluten films. RSC Adv. 2012, 2, 11908–11914. 10.1039/c2ra21812g.

[ref37] Rasheed F.; Hedenqvist M. S.; Kuktaite R.; Plivelic T. S.; Gällstedt M.; Johansson E. Mild gluten separation–A non-destructive approach to fine tune structure and mechanical behavior of wheat gluten films. Ind. Crops Prod. 2015, 73, 90–98. 10.1016/j.indcrop.2015.04.007.

[ref38] Kuktaite R.; Newson W. R.; Rasheed F.; Plivelic T. S.; Hedenqvist M. S.; Gällstedt M.; Johansson E. Monitoring Nanostructure Dynamics and Polymerization in Glycerol Plasticized Wheat Gliadin and Glutenin Films: Relation to Mechanical Properties. ACS Sustainable Chem. Eng. 2016, 4, 2998–3007. 10.1021/acssuschemeng.5b01667.

[ref39] Rasheed F.; Newson W. R.; Plivelic T. S.; Kuktaite R.; Hedenqvist M. S.; Gallstedt M.; Johansson E. Structural architecture and solubility of native and modified gliadin and glutenin proteins: non-crystalline molecular and atomic organization. RSC Adv. 2014, 4, 2051–2060. 10.1039/C3RA45522J.

[ref40] Rombouts I.; Lagrain B.; Brunnbauer M.; Koehler P.; Brijs K.; Delcour J. A. Identification of Isopeptide Bonds in Heat-Treated Wheat Gluten Peptides. J. Agric. Food Chem. 2011, 59, 1236–1243. 10.1021/jf103579u. 21235244

[ref41] Ye X.; Capezza A. J.; Gowda V.; Olsson R. T.; Lendel C.; Hedenqvist M. S. High-Temperature and Chemically Resistant Foams from Sustainable Nanostructured Protein. Adv. Sustainable Syst. 2021, 5, 2100063 10.1002/adsu.202100063.

[ref42] Wu Q.; Yu S.; Kollert M.; Mtimet M.; Roth S. V.; Gedde U. W.; Johansson E.; Olsson R. T.; Hedenqvist M. S. Highly Absorbing Antimicrobial Biofoams Based on Wheat Gluten and Its Biohybrids. ACS Sustainable Chem. Eng. 2016, 4, 2395–2404. 10.1021/acssuschemeng.6b00099.

[ref43] Olabarrieta I.; Cho S. W.; Gällstedt M.; Sarasua J. R.; Johansson E.; Hedenqvist M. S. Aging properties of films of plasticized vital wheat gluten cast from acidic and basic solutions. Biomacromolecules 2006, 7, 1657–1664. 10.1021/bm0600973. 16677051

[ref44] Cuq B.; Gontard N.; Guilbert S. Proteins as Agricultural Polymers for Packaging Production. Cereal Chem. J. 1998, 75, 1–9. 10.1094/CCHEM.1998.75.1.1.

[ref45] Morris H.; Murray R. Medical textiles. Text. Prog. 2020, 52, 1–127. 10.1080/00405167.2020.1824468.

[ref46] Capezza A. J.; Lundman M.; Olsson R. T.; Newson W. R.; Hedenqvist M. S.; Johansson E. Carboxylated Wheat Gluten Proteins: A Green Solution for Production of Sustainable Superabsorbent Materials. Biomacromolecules 2020, 21, 1709–1719. 10.1021/acs.biomac.9b01646. 31899621

[ref47] Capezza A. J.; Cui Y.; Numata K.; Lundman M.; Newson W. R.; Olsson R. T.; Johansson E.; Hedenqvist M. S. Superabsorbent Polymers: High Capacity Functionalized Protein Superabsorbents from an Agricultural Co-Product: A Cradle-to-Cradle Approach (Adv. Sustainable Syst. 9/2020). Adv. Sustainable Syst. 2020, 4, 2070018 10.1002/adsu.202070018.

[ref48] Capezza A. J.; Robert E.; Lundman M.; Newson W. R.; Johansson E.; Hedenqvist M. S.; Olsson R. T. Extrusion of Porous Protein-Based Polymers and Their Liquid Absorption Characteristics. Polymers 2020, 12, 459 10.3390/polym12020459. 32079125 PMC7077648

