# Supplementary material for: Innovative Green Way to Design Biobased Electrospun Fibers from Wheat Gluten and These Fibers’ Potential as Absorbents of Biofluids
Source: ACS Environ Au. 2022 Jan 21;2(3):232–41. doi: 10.1021/acsenvironau.1c00049 (PMC10125173; doi:10.1021/acsenvironau.1c00049)

Supporting Information

Innovative Green Way to Design Bio-based Electrospun Fibers from Wheat Gluten and these Fibers Potential as Absorbents of Biofluids

Faraz Muneer,[a] Mikael S. Hedenqvist,[b] Stephen Hall,[c] Ramune Kuktaite,[a*]

[a]Department of Plant Breeding, Swedish University of Agricultural Sciences, Box 190, SE-23422 Lomma, Sweden

[b]KTH Royal Institute of Technology, Fiber and Polymer Technology Department, SE-100 44 Stockholm, Sweden

[c]Solid Mechanics, Lund Institute of advanced Neutron and X-ray Science (LINXS), Lund University, Box 117, SE-221 00 Lund, Sweden

*Corresponding author: ramune.kuktaite@slu.se

Supporting Information. FT-IR spectra of all the WG fibers and heat-treated fiber samples (Table S1, Figure S1) and T-WGF15 fibers stability in PBS solution (Figure S2)

Table S1. FTIR de-convoluted absorbance spectra and relative amounts of different secondary structures present in gluten fiber samples.

Peak	Assignment	WGF15	T-WGF15	WGF20	T-WGF20
1607 (1.0)	β-sheets	2.5	1.9	14	1.5
1615 (1.5)	β-sheets	8.78	4.6	5.3	5.7
1624 (1.1)	β-sheets, strongly hydrogen bonded peptide groups	---	10.4	14.8	15.2
1636 (3.2)	β-sheets, weakly hydrogen bonded peptide groups	31.1	27.3	---	26.8
1646 (3.1)	Unordered	18.7	---	42.8	---

1652 (2.1)	α-helices and random coils	0.6	24.3	9.1	1.2
1661 (2.9)	α-helices	13.6	19.1	1.4	36.6
1673 (4.5)	β-turns	10.1	7.9	18.6	9.0
1684 (3.4)	β-sheets, weakly hydrogen bonded peptide groups	12.1	3.4	5.4	3.1
1693 (1.0)	β-turns	2.3	0.9	1.1	0.7

Figure S1. Gaussian curve fittings of individual FT-IR profile of a) WGF15, b) T-WGF15, c) WGF20 and d) T-WGF20.

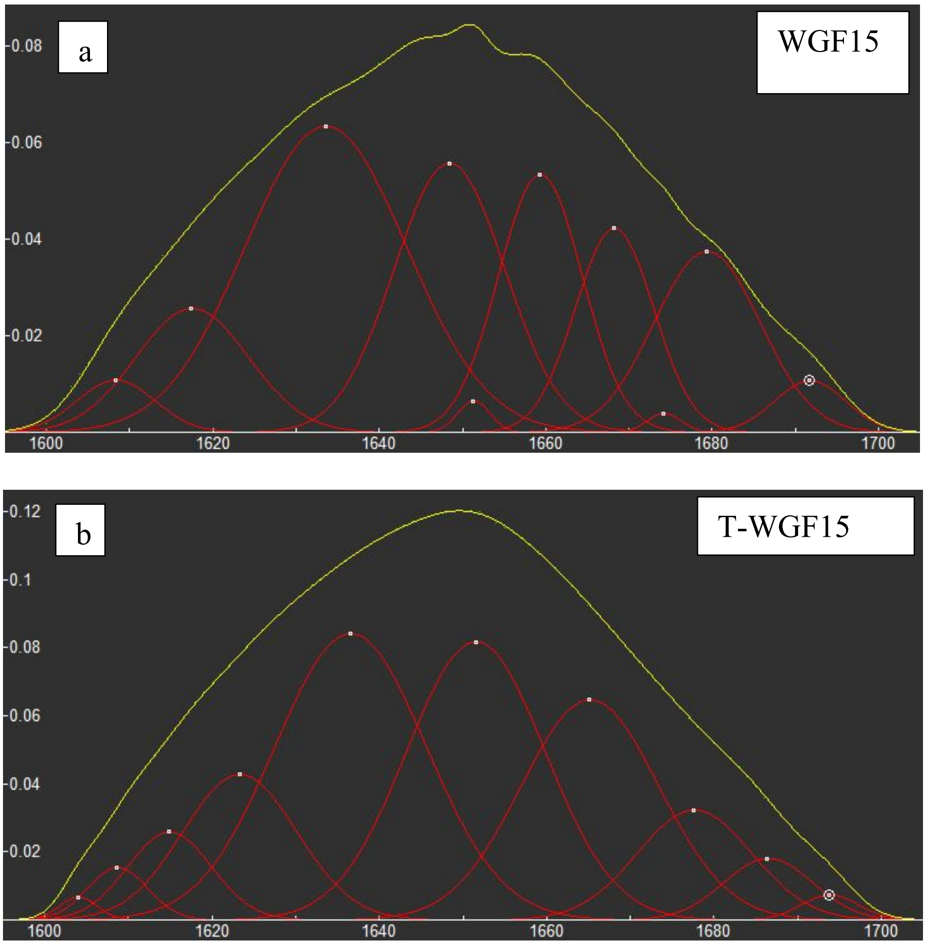

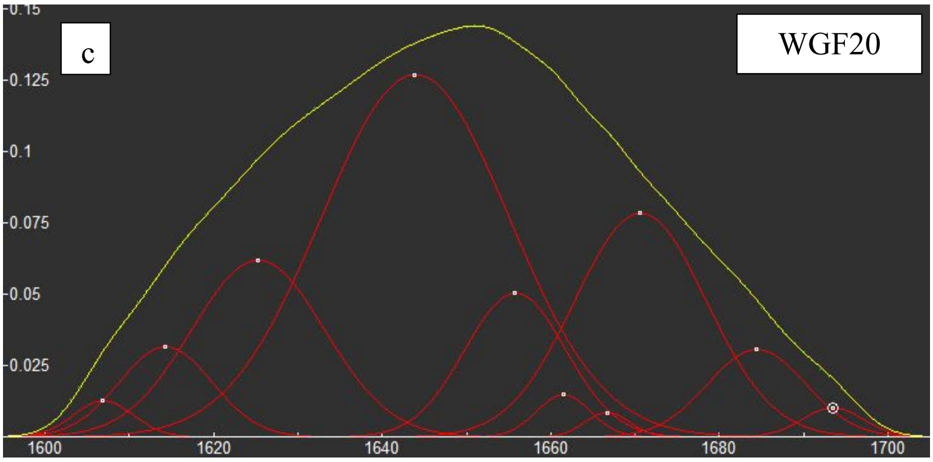

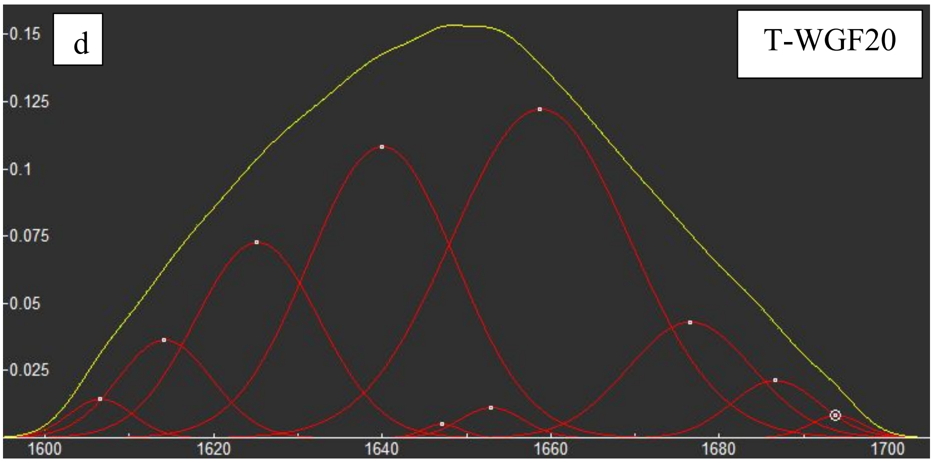

Figure S2. The T-WGF15 immersed in PBS buffer solution after 1 h.

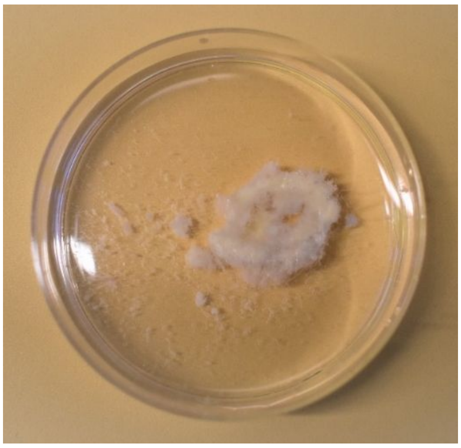

Supplement: Supplementary file 1 — vg1c00049_si_001.pdf [file vg1c00049_si_001.pdf]
